# Transmission Dynamics of Carbapenem-Resistant *Klebsiella pneumoniae* Sequence Type 11 Strains Carrying Capsular Loci KL64 and *rmpA*/*rmpA2* Genes

**DOI:** 10.3389/fmicb.2021.736896

**Published:** 2021-10-07

**Authors:** Yingying Kong, Qingyang Sun, Hangfei Chen, Mohamed S. Draz, Xinyou Xie, Jun Zhang, Zhi Ruan

**Affiliations:** ^1^Department of Clinical Laboratory, Sir Run Run Shaw Hospital, Zhejiang University School of Medicine, Hangzhou, China; ^2^Department of Clinical Laboratory, No. 903 Hospital of PLA Joint Logistic Support Force, Hangzhou, China; ^3^Department of Medicine, Case Western Reserve University School of Medicine, Cleveland, OH, United States

**Keywords:** *Klebsiella pneumoniae*, carbapenem resistance, outbreak, whole-genome sequencing, single-nucleotide polymorphisms, genomic surveillance, hypervirulence plasmid

## Abstract

The presence and dissemination of carbapenem-resistant *Klebsiella pneumoniae* (CRKP) often cause life-threatening infections worldwide, but the therapeutic option is limited. In this study, whole-genome sequencing (WGS) was applied to assess the epidemiological characteristics and transmission dynamics of CRKP isolates recovered from two fetal outbreaks of nosocomial infections. Between April 2016 and March 2018, a total of 70 isolates of *K. pneumoniae* were collected from sterile samples in a tertiary hospital in Hangzhou, China. The minimal inhibitory concentrations (MICs) of 21 antimicrobial agents were determined using the broth microdilution methods. Pulsed-field gel electrophoresis (PFGE) was performed on 47 CRKP isolates, and 16 clonally related isolates were further characterized by Illumina sequencing. In addition, the complete genome sequences of three representative isolates (KP12, KP36, and KP37) were determined by Oxford Nanopore sequencing. The *K. pneumoniae* isolates were recovered from patients diagnosed with pulmonary infection, cancer, or encephalopathy. For all CRKP isolates, PFGE separated three clusters among all strains. The most predominant PFGE cluster contained 16 isolates collected from patients who shared close hospital units and represented a potential outbreak. All 16 isolates showed an extremely high resistance level (≥87.5%) to 18 antimicrobials tested but remain susceptible to colistin (CST). Multiple antimicrobial resistance and virulence determinants, such as the carbapenem resistance gene *bla*_KPC-2_, and genes encoding the virulence factor aerobactin and the regulator of the mucoid phenotype (*rmpA* and *rmpA2*), were observed in the 16 CRKP isolates. These isolates belonged to sequence type 11 (ST11) and capsular serotype KL64. A core genome single nucleotide polymorphism (cgSNP)-based phylogenetic analysis indicated that the 16 CRKP isolates could be partitioned into two separate clades (≤15 SNPs), suggesting the two independent transmission scenarios co-occurred. Moreover, a high prevalence of IncFIB/IncHI1B type virulence plasmid with the *iroBCDN* locus deleted, and an IncFII/IncR type *bla*_KPC-2_-bearing plasmid was co-harbored in ST11-KL64 CRKP isolates. In conclusion, our data indicated that the nosocomial dissemination of ST11-KL64 CRKP clone is a potential threat to anti-infective therapy. The development of novel strategies for surveillance, diagnosis, and treatment of this high-risk CRKP clone is urgently needed.

## Introduction

*Klebsiella pneumoniae*, as an increasingly important human pathogen, represents increasingly multidrug-resistance, particularly to carbapenems and the third-generation cephalosporins (Navon-Venezia et al., 2017). Carbapenem-resistant *K. pneumoniae* (CRKP) is widely reported as a multidrug-resistant bacteria and associated with high morbidity and mortality rates (Wyres et al., 2020a). CRKP can produce carbapenem-hydrolyzing enzymes to hydrolyze carbapenemase and eventually nullify the effectiveness of the last-resort antibiotics. In 1996, the first *K. pneumoniae* carbapenemase (KPC) enzyme, encoded by the *bla*_KPC_ gene, was found in *K. pneumoniae* (Yigit et al., 2001). Subsequently, other carbapenemase genes have emerged, such as *bla*_NDM_, *bla*_OXA-48_, *bla*_VIM_, and *bla*_IMP_ (Fukigai et al., 2007; Perez-Vazquez et al., 2019; Lu et al., 2020; Nishida et al., 2020). The KPC-producing isolates are always reported to be associated with nosocomial outbreaks worldwide. In China, outbreaks of CRKP isolates mainly carried the *bla*_KPC-2_ or *bla*_NDM-1_ gene and can be classified as sequence type (ST)11 by multilocus sequence typing (MLST; Gu et al., 2018; Zhang et al., 2020a). Unfortunately, the KPC-producing isolates are resistant to almost all β-lactams and β-lactamase inhibitors, which significantly limits treatment options and eventually leads to high mortality rates, especially among inpatients with prolonged hospitalization (Jiang et al., 2015; Gu et al., 2018; Sui et al., 2018; Zhang et al., 2020a).

Compared with the classic *K. pneumoniae* (cKP), hypervirulent *K. pneumoniae* (hvKP) causes more severe infections, such as liver abscesses, endophthalmitis, meningitis, and pneumonia, and displays a higher level of susceptibility to the antimicrobial agents (Wang et al., 2018; Russo and Marr, 2019; Choby et al., 2020). The hvKP is undergoing global dissemination and has been confirmed to be highly associated with several virulence factors as the hallmarks, including the regulator of the mucoid phenotype (encoded by the *rmpA* gene), the regulator of mucoid phenotype 2 (*rmpA2*), aerobactin (*iucABCD*, *iutA*), salmochelin (*iroBCDN*), and metabolite transporter (*peg-344*), which were typically co-located on a classic pLVPK-like virulence plasmid (Wyres et al., 2020b). However, hvKP strains are becoming increasingly resistant to antimicrobials, including carbapenems (Feng et al., 2018; Gu et al., 2018). Moreover, the combination of carbapenem resistance and hypervirulence significantly reduces the efficacy of antimicrobial agents to treat the life-threatening infections caused by carbapenem-resistant hvKP (CR-hvKP). Therefore, they represent an extremely severe health challenge and public concern. Consequently, it is urgent to investigate the genomic characteristics of CRKP to prevent, diagnose, and treat *K. pneumoniae* infections.

Rapid advances in whole-genome sequencing (WGS) technology and bioinformatics tools can facilitate understanding the spread of *K. pneumoniae*, including the identification of transmission routes, evolutionary patterns, and antimicrobial resistance mechanisms (Klemm et al., 2018; Schurch et al., 2018). Due to the advances in next-generation sequencing platforms, the cost of WGS continues to decrease. Moreover, WGS can detect minor genomic differences between isolates for its high resolution, limited in traditional molecular typing techniques, such as pulsed-field gel electrophoresis (PFGE) and MLST.

In this study, a total of 70 *K. pneumoniae* isolates were collected from patients during their hospitalizations in a tertiary hospital in China. The 16 KPC-2-producing CRKP isolates, represented a hospital outbreak based on antimicrobial susceptibility testing and PFGE, were further subjected to WGS analysis. The genomic epidemiological characteristics, the transmission route of this outbreak, and the genetic features of the virulence plasmids and *bla*_KPC-2_-bearing plasmids were investigated.

## Materials and Methods

### Patients and Isolates

Seventy *K. pneumoniae* isolates, recovered from patients in severe conditions or long-term hospitalization, were collected from a tertiary hospital in Hangzhou, Zhejiang province, China, between April 2016 and March 2018. The quick Sequential Organ Failure Assessment (qSOFA) score and the Confusion, Urea, Respiratory rate, Blood pressure plus age≥65years (CURB-65) score were used to help determine patients with severe illness. An inpatient hospitalization lasting 14 days or more was considered a long hospital stay. All isolates were cultured from the sputum, urine, blood, excreta, catheters, feces, and cerebrospinal fluids specimens. Demographic data, such as gender, age, department of hospitalization, clinical diagnosis, outcome, time of admission, time of discharge, and length of hospital stay, were extracted from the patient administration system. This study was approved by the local Research Ethics Committee of Sir Run Run Shaw Hospital, Zhejiang University School of Medicine. All isolates were generated as part of routine clinical laboratory procedures, and no identifiable patient information was collected.

### Bacterial Identification and Antimicrobial Susceptibility

Bacterial identification was performed using VITEK 2 (bioMérieux, Marcy-l’Étoile, France) and Matrix-assisted laser desorption/ionization–time-of-flight mass spectrometry (MALDI-TOF-MS, Bruker, Billerica, MA, United States). Antimicrobial susceptibility testing was carried out for all isolates using the broth microdilution methods. The susceptibility breakpoint was interpreted according to Clinical and Laboratory Standards Institute (CLSI) 2020 guidelines or European Committee on Antibiotic Susceptibility Testing (EUCAST) 10.0 guidelines. The list of tested antimicrobial agents includes the following: aztreonam (ATM), fosfomycin (FOF), ertapenem (ETP), ceftazidime (CAZ), cefepime (FEP), cefoperazone-sulbactam (SCF), cefoxitin (FOX), levofloxacin (LVX), ciprofloxacin (CIP), amikacin (AMK), tetracycline (TET), tigecycline (TGC), minocycline (MH), colistin (CST), cefotaxime (CTX), meropenem (MEM), imipenem (IPM), gentamicin (GEN), trimethoprim-sulfamethoxazole (SXT), piperacillin (PRL), and piperacillin-tazobactam (PRL/TZP). *Escherichia coli* ATCC 25922 was used as a quality control strain for antimicrobial susceptibility testing.

### Pulsed-Field Gel Electrophoresis

All the 47 CRKP isolates were characterized by PFGE. Genomic DNA of isolates was digested overnight with XbaI restriction enzyme (Sangon, Shanghai, China), and the DNA fragments were subjected to electrophoresis in 1% agarose III (Sangon) with a CHEF apparatus (CHEF Mapper XA; Bio-Rad, Hercules, CA, United States). The electrophoresis conditions were 14°C and 6V/cm with alternating pulses at a 120° angle with a 5–35s pulse time gradient for 22h. *Salmonella enterica* serotype Braenderup H9812 was used as a molecular size marker, covering the fragment ranges generated by *K. pneumoniae*. The PFGE patterns were analyzed by BioNumerics 7.0 software (Applied Maths, Sint-MartensLatem, Belgium) with the Dice similarity index. Interpretation is based on the criteria proposed by Tenover et al. (1995), that is, two isolates shared no more than three band differences of PFGE patterns are deemed as the same clone.

### Whole-Genome Sequencing

Total DNA was extracted from the 16 CRKP isolates using a QIAamp DNA Mini Kit (Qiagen, Valencia, CA, United States) and fragmented by sonication using a Covaris M220 sonicator (Covaris, Woburn, MA, United States). Genomic libraries were prepared with an average insert size of 350bp using a TruSeq DNA Sample Prep kit (Illumina, San Diego, CA, United Staets) and sequenced using the Illumina NovaSeq 6,000 platform (Illumina, San Diego, CA, United States) with the 150bp paired-end protocol. Furthermore, the genomic DNA of three representative isolates (KP12, KP36, and KP37) out of the 16 CRKP isolates was also sequenced on the long-read Oxford Nanopore MinION platform (Nanopore Technologies, Oxford, United Kingdom). The derived short Illumina reads and long MinION reads were assembled using Unicycler v0.4.8 software (Wick et al., 2017).

The genome annotation was performed using the NCBI Prokaryotic Genome Annotation Pipeline (PGAP; Tatusova et al., 2016). Acquired antimicrobial resistance genes and virulence genes were identified using ResFinder 4.1 and VFDB 2019 databases, respectively, with a 90% threshold for gene identification and a 60% minimum length to respective database entries. *In silico* MLST analysis was performed by BacWGSTdb 2.0 server (Ruan and Feng, 2016; Ruan et al., 2020b; Feng et al., 2021). The type of capsule and lipopolysaccharide serotype of *K. pneumoniae* was conducted using Kaptive v0.6.1 (Wick et al., 2018).

### Single-Nucleotide Polymorphisms and Phylogenetic Analysis

The ST11 *K. pneumoniae* strain KP58, another ST11 CRKP isolate recovered in Hangzhou, China, was used as a reference sequence to identify genomic variations between 16 CRKP isolates. Bacterial core genome single nucleotide polymorphism (cgSNP) was analyzed using the BacWGSTdb 2.0 server (Ruan and Feng, 2016; Feng et al., 2021). SNPs in the core genome and the removal of recombination regions from SNP alignments were predicted using Snippy v4.4.5. The output was used to construct a phylogenetic tree, with 1,000 bootstraps, under the general time-reversible (GTR) model with RAxML v8.2.12, and also used to identify the pairwise SNP distances using snp-dist v0.6.3 (Stamatakis, 2014). Isolates were considered the same outbreak if the threshold for SNPs distance≤18 (Schurch et al., 2018). Visualization and annotation of the phylogenetic tree and the presence of antimicrobial resistance genes and plasmid-borne virulence genes were performed by the Interactive Tree Of Life (iTOL) v5 webserver (Letunic and Bork, 2021). Sequence comparison of virulence plasmids and *bla*_KPC-2_-bearing plasmids was conducted using BLAST Ring Image Generator (BRIG) and Easyfig (Alikhan et al., 2011; Sullivan et al., 2011).

### Accession Number

The genome sequences of the 16 CRKP isolates were deposited in the NCBI GenBank database under the BioProject accession numbers PRJNA553055.

## Results

### Clinical Characteristics of *K. pneumoniae* Isolates

From April 2016 to March 2018, a total of 70 *K. pneumoniae* isolates were cultured from 68 inpatients in a tertiary hospital in Hangzhou. The mean age of the inpatients was 70.5±17.3years. Among the 68 inpatients, 47 (69.12%) inpatients were males, and 21 (30.88%) were females. These clinical isolates were cultured from sputum (28/70, 40.00%), urine (20/70, 28.57%), blood (12/70, 17.14%), excreta (7/70, 10.00%), catheters (1/70, 1.43%), feces (1/70, 1.43%), and cerebrospinal fluids (1/70, 1.43%). The inpatients were diagnosed as pulmonary infection (19/68, 27.94%), cancer (9/68, 13.24%), encephalopathy (23/68, 33.82%), upper gastrointestinal bleeding (2/68, 2.94%), abdominal pain (1/68, 1.47%), hydronephrosis (1/68, 1.47%), cardiopulmonary arrest (1/68, 1.47%), chronic sinusitis (1/68, 1.47%), perianal abscess (1/68, 1.47%), diabetes (1/68, 1.47%), and nine (13.24%) inpatients suffered external injury or had a history of surgery. Among the 70 *K. pneumoniae* isolates, 47 isolates showed a carbapenem resistance phenotype, with a rate of 67.14%.

### Comparison of Outbreak Isolates According to PFGE and WGS-Based SNPs

**Figure 1:**
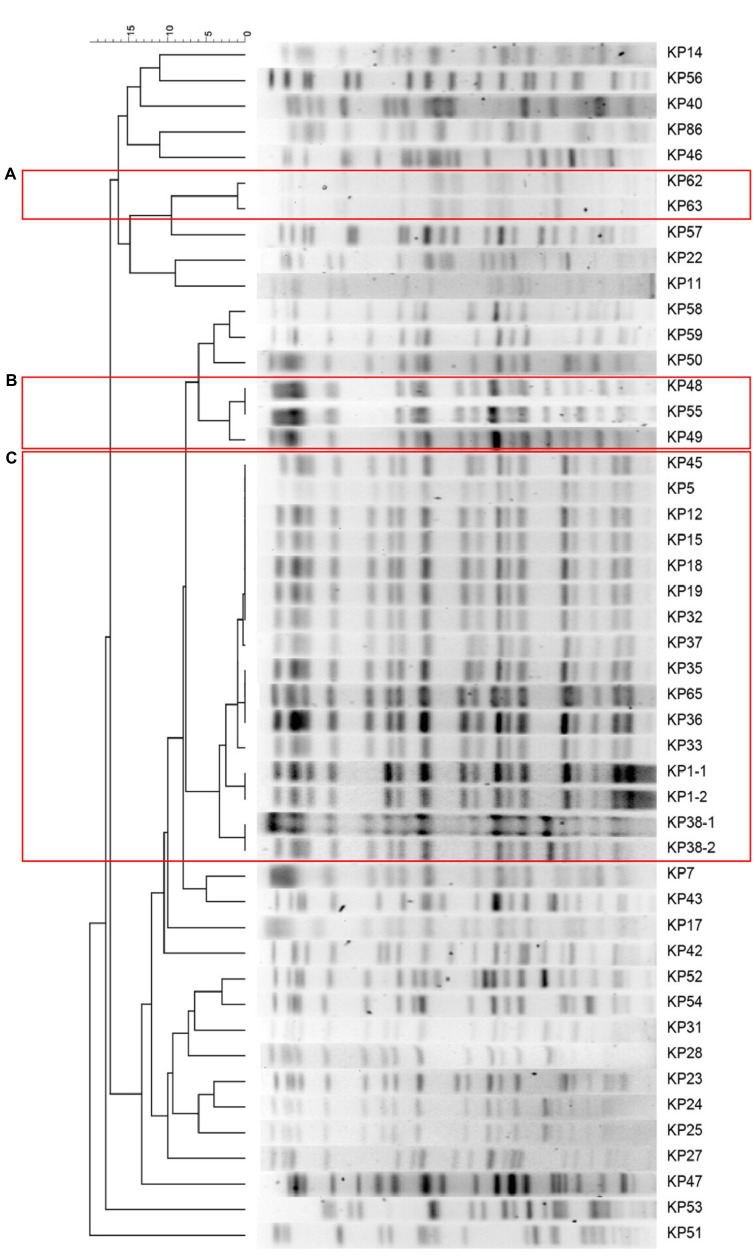
Clustering of the 16 carbapenem-resistant *Klebsiella pneumoniae* (CRKP) isolates based on pulsed-field gel electrophoresis (PFGE) profiles. The information of strain ID is listed to the left of the patterns. The three predominant PFGE cluster **(A)**, **(B)**, and **(C)** are represented by red rectangles.

Based on the PFGE typing results of 47 CRKP isolates, three clusters were observed according to the similarity between PFGE banding patterns (Figure 1). The dominant PFGE cluster C contained 16 isolates and represented a putative outbreak in the hospital for similar PFGE patterns (differed by <three bands). The PFGE cluster A and B included two and three isolates, respectively. In contrast, the remaining 26 isolates showed sporadic PFGE patterns.

The draft genomes of the 16 CRKP isolates, presented in PFGE cluster C, were determined by whole-genome sequencing. All the isolates harbored *bla*_KPC-2_ gene. According to the MLST scheme of *K. pneumoniae*, all the 16 KPC-2-producing CRKP isolates were identified to be ST11 and shared the same capsular serotype KL64.

**Figure 2:**
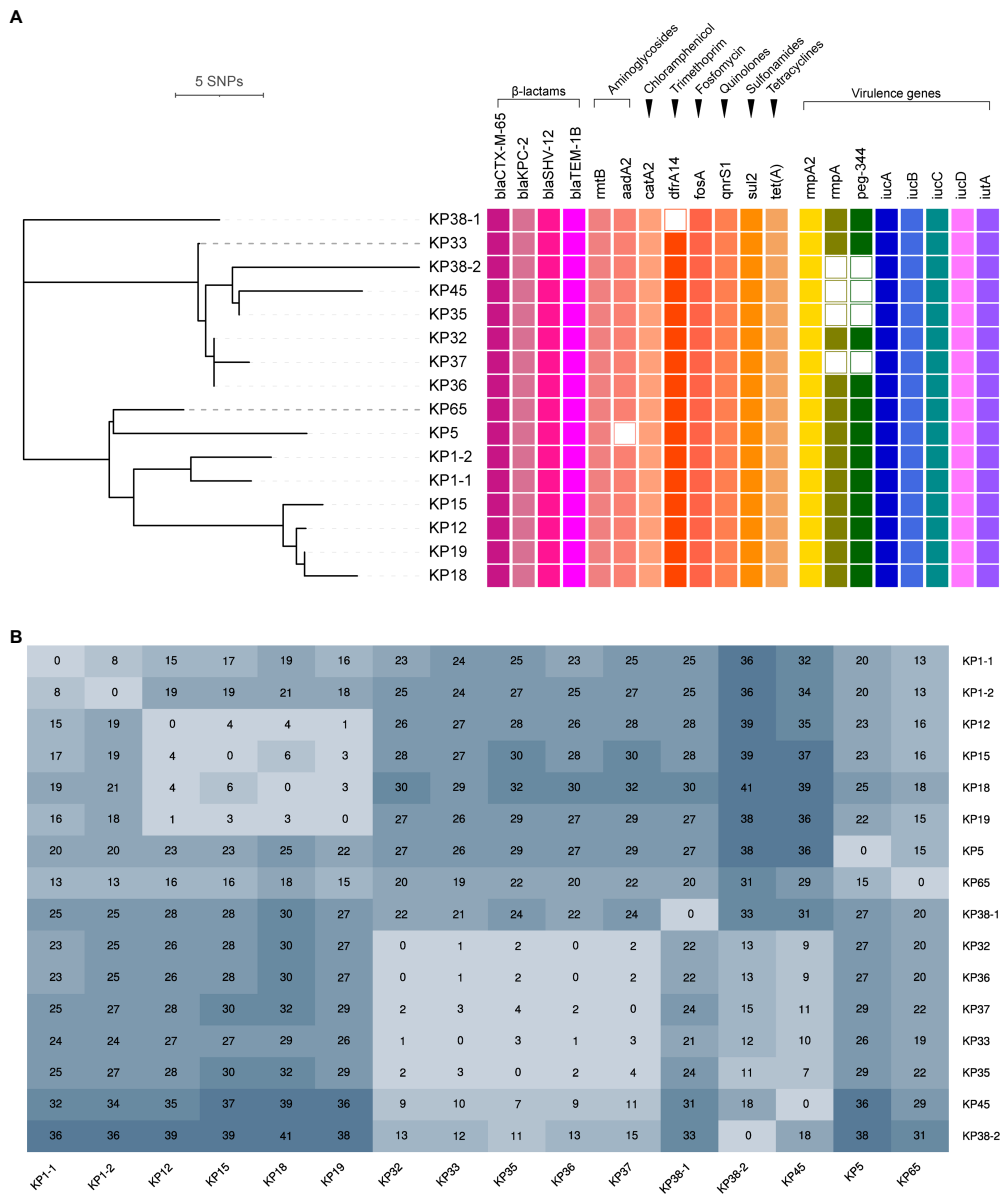
Phylogenetic analysis of 16 *K. pneumoniae* carbapenemase (KPC)-2-producing ST11-KL64 CRKP isolates. **(A)** Recombination-filtered core genome phylogeny and the distribution of antimicrobial resistance genes and virulence genes in the CRKP isolates. The cell in different colors represents the presence of the gene, while the blank cell represents the absence of the gene. **(B)** The single nucleotide polymorphisms (SNPs) numbers between each isolate.

Phylogenetic analysis indicated that all the ST11-KL64 CRKP isolates were partitioned into two clades (Figure 2A). Clade 1 contained seven isolates (KP32, KP33, KP35, KP36, KP37, KP45, and KP38-2), and clade 2 comprised four isolates (KP12, KP15, KP18, and KP19), while the remaining five isolates (KP38-1, KP1-1, KP1-2, KP5, and KP65) were singletons. The number of SNPs separating ST11-KL64 CRKP isolates ranged from 0 to 41 after removing recombination regions (Figure 2B). Concerning the different variants among the 16 isolates, the internal members of each clade were closely related (≤18 SNPs), and a high diversity between the two clades was also observed. The differences in the internal isolates of clade 1 and 2 ranged from 0 to 18 SNPs and 1 to 6 SNPs, respectively, representing two independent outbreaks. It is noteworthy that only eight SNPs were detected between KP1-1 and KP1-2 (recovered from the same patient), suggesting they were the variants of the same clone. In comparison, 33 SNPs were observed between KP38-1 and KP38-2, implying that the patient Pa38 were infected with two different clones of CRKP. The detailed information for SNPs detected in 16 *K. pneumoniae* strains is shown in Supplementary Table S1.

### Clinical and Microbiological Characteristics of the 16 ST11-KL64 CRKP Isolates

**Figure 3:**
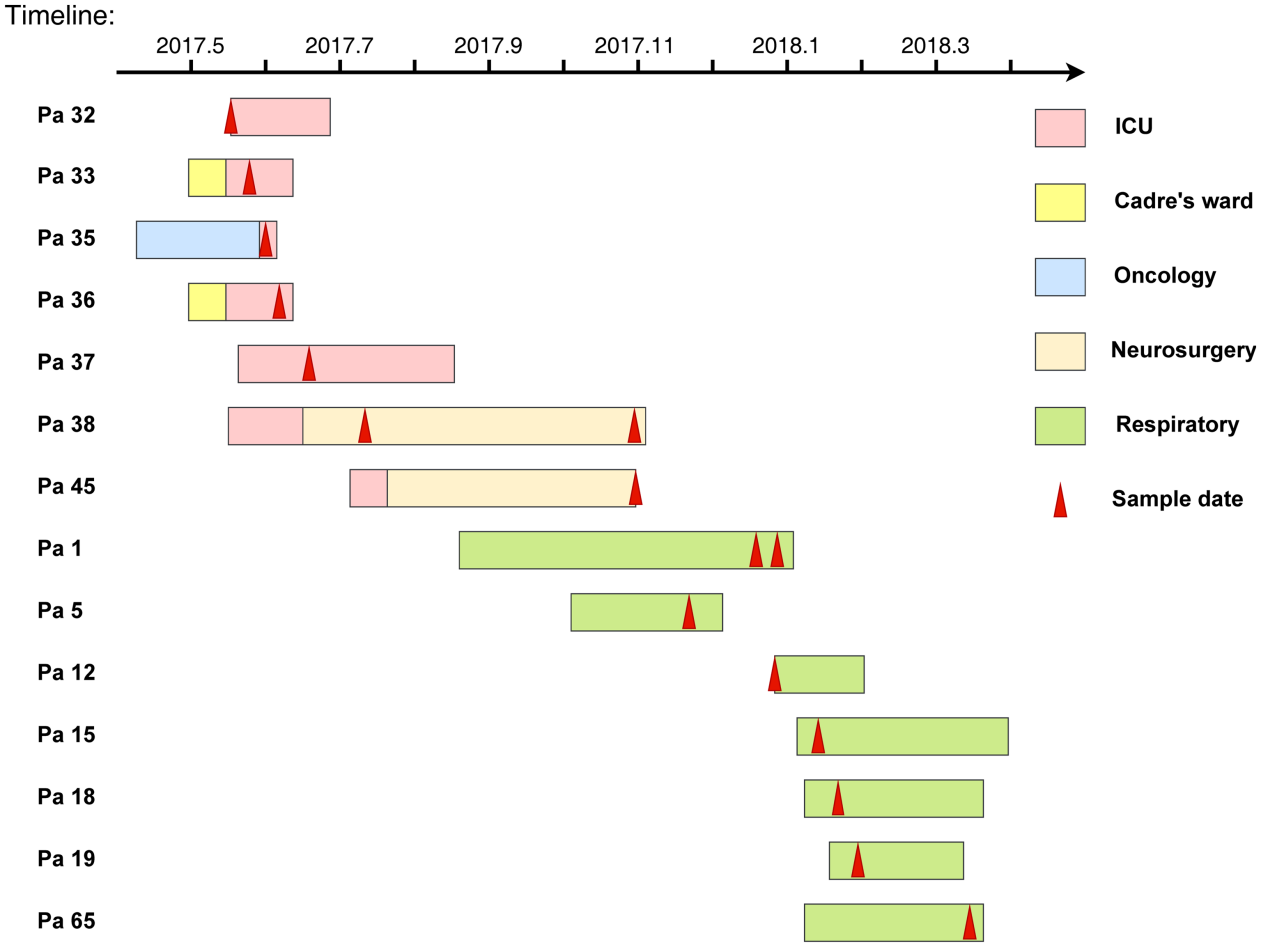
The timelines of inpatients harboring KPC-2-producing ST11-KL64 CRKP isolates in the clinical setting. The rectangle on the timeline represents the admitting’s duration of the patients. Different wards are shown in different colors, and the red arrows indicate sampling dates.

Demographic and clinical characteristics and the timeline of inpatients with 16 KPC-2-producing ST11-KL64 CRKP clinical cultures are shown in Table 1 and Figure 3. The 16 strains were isolated from 14 separate patients admitted into the respiratory (KP1-1, KP1-2, KP5, KP12, KP15, KP18, KP19, and KP65), intensive care unit (ICU; KP32, KP33, KP35, KP36, and KP37), and neurosurgery (KP38-1, KP38-2, and KP45). Isolate KP32, isolated from patient 32 (Pa 32) on May 15, 2017, represented the first case of the first outbreak. In the next 34 days, another four CRKP isolates (KP33, KP35, KP36, and KP37) were cultured from the sputum and blood specimens of four patients in the ICU. About 22 days later, KP38-1 and KP38-2 were isolated from the blood and sputum of the same patient (Pa38) admitted into neurosurgery with a 112-day interval, respectively. KP45 was isolated from Pa45 in the same ward on the same day of KP38-2 isolated. Between November 2017 and January 2018, another seven inpatients were continuously confirmed to have CRKP infection in the respiratory ward. Finally, 10 patients gradually recovered among the 14 patients involved in this outbreak, and four died of CRKP infection.

**Table 1:** Characteristics and outcomes of the 14 inpatients involved in the KPC-2-producing ST11-KL64 CRKP isolates outbreaks.

Patient	Isolate number	Age (years)	Gender	Department	Diagnosis	Outcome	Isolate date	Isolate type
Pa1	KP1-1	66	Female	Respiratory	History of pancreatic malignancy	Died	19/12/2017	Blood
	KP1-2	66	Female	Respiratory	History of pancreatic malignancy	Died	28/12/2017	Feces
Pa5	KP5	57	Male	Respiratory	Pulmonary malignancy	Discharged	20/11/2017	Blood
Pa12	KP12	53	Male	Respiratory	Pulmonary malignancy	Discharged	25/12/2017	Urine
Pa15	KP15	91	Male	Respiratory	Pulmonary infection	Discharged	13/1/2018	Urine
Pa18	KP18	73	Male	Respiratory	Pulmonary infection	Discharged	21/1/2018	Sputum
Pa19	KP19	93	Female	Respiratory	Pulmonary infection, heart failure	Discharged	29/1/2018	Urine
Pa32	KP32	61	Male	ICU	Severe craniocerebral injury	Discharged	15/5/2017	Sputum
Pa33	KP33	96	Male	ICU	Pulmonary malignancy, respiratory failure	Died	25/5/2017	Sputum
Pa35	KP35	60	Female	ICU	Pulmonary infection, intracranial space-occupied	Discharged	1/6/2017	Sputum
Pa36	KP36	96	Male	ICU	Pulmonary malignancy, respiratory failure	Died	7/6/2017	Blood
Pa37	KP37	36	Male	ICU	Cardiopulmonary arrest	Discharged	18/6/2017	Blood
Pa38	KP38-1	92	Female	Neurosurgery	Aspiration pneumonia, hypertension	Died	10/7/2017	Urine
	KP38-2	92	Female	Neurosurgery	Sequelae of cerebral infarction, Aspiration pneumonia	Died	30/10/2017	Blood
Pa45	KP45	83	Female	Neurosurgery	Post-traumatic syndrome, cerebral hemorrhage	Discharged	30/10/2017	Urine
Pa65	KP65	73	Male	Respiratory	Pulmonary infection	Discharged	14/3/2018	Excreta

ICU, intensive care unit.

### Antimicrobial Susceptibility Testing

All 16 ST11-KL64 CRKP isolates were multidrug-resistant (Table 2). According to the antimicrobial susceptibility testing data, all the *bla*_KPC_-carrying *K. pneumoniae* isolates displayed resistance to ATM (MIC≥32mg/L), ETP (MIC≥256mg/L), CAZ (MIC≥128mg/L), FEP (MIC≥256mg/L), SCF (MIC>256mg/L), FOX (MIC≥128mg/L), LVX (MIC≥16mg/L), CIP (MIC≥16mg/L), TET (MIC≥256mg/L), MH (MIC≥16mg/L), CTX (MIC≥64mg/L), MEM (MIC=128mg/L), IPM (MIC≥64mg/L), GEN (MIC≥256mg/L), PRL (MIC>512mg/L), and PRL/TZP (MIC≥512mg/L). Moreover, a high level of resistance (≥87.5%) was observed to AMK (MIC >256mg/L, except for KP5) and SXT (MIC=16mg/L, except for KP38-1 and KP38-2). Additionally, resistance to FOF and TGC was observed in 4 (25.00%) and 3 (18.75%) isolates, respectively, and colistin resistance was detected only in one isolate KP1-2 (6.25%). Compared to the 16 ST11-KL64 CRKP isolates, the remaining 31 CRKP isolates showed relatively more susceptibility to most antimicrobials, especially for tetracycline and minocycline, but higher resistant rates of fosfomycin and trimethoprim-sulfamethoxazole. Most of the remaining 31 CRKP isolates also maintained high resistance (resistance rate>80.65%) to the aztreonam, ertapenem, ceftazidime, cefepime, cefoperazone-sulbactam, cefoxitin, levofloxacin, ciprofloxacin, amikacin, cefotaxime, meropenem, imipenem, gentamicin, trimethoprim-sulfamethoxazole, piperacillin, and piperacillin-tazobactam (Supplementary Table S2).

**Table 2:** Minimal inhibitory concentrations (MICs) of the 16 KPC-2-producing ST11-KL64 CRKP isolates to different antimicrobials.

Isolate	ATM^a^	FOF	ETP	CAZ	FEP	SCF	FOX	LVX	CIP	AMK	TET	TGC	MH	CST	CTX	MEM	IPM	GEN	SXT	PRL	PRL/TZP
KP1-1	32^b^	16	>256	>256	256	>256	256	64	128	>256	>256	8	64	0.03	64	128	128	>256	16	>512	>512
KP1-2	32	32	>256	256	256	>256	128	128	128	>256	>256	8	64	64	128	128	128	>256	16	>512	512
KP5	128	64	>256	>256	256	>256	>256	128	128	1	>256	4	64	0.03	>256	128	128	>256	16	>512	>512
KP12	128	128	256	>256	256	>256	256	32	64	>256	256	2	32	0.06	>256	128	128	256	16	>512	>512
KP15	128	32	>256	>256	256	>256	>256	64	128	>256	256	4	64	0.03	>256	128	32	>256	16	>512	>512
KP18	128	256	>256	>256	256	>256	>256	64	64	>256	256	4	32	0.06	>256	128	128	>256	16	>512	>512
KP19	128	256	>256	>256	256	>256	256	32	64	>256	>256	4	32	0.06	>256	128	128	>256	16	>512	>512
KP32	128	16	>256	>256	256	>256	>256	64	128	>256	>256	4	64	0.03	>256	128	128	>256	16	>512	>512
KP33	128	32	>256	>256	512	>256	>256	128	128	>256	>256	4	64	0.03	>256	128	128	>256	16	>512	>512
KP35	128	32	>256	>256	256	>256	>256	64	128	>256	>256	4	64	0.125	>256	128	128	>256	16	>512	>512
KP36	128	32	256	>256	256	>256	256	64	128	>256	>256	4	64	2	256	128	128	>256	16	>512	>512
KP37	128	32	>256	>256	256	>256	>256	128	128	>256	>256	4	32	0.03	>256	128	128	>256	16	>512	>512
KP45	128	32	256	256	256	>256	256	16	16	>256	256	4	16	0.03	256	128	128	>256	16	>512	512
KP65	128	64	>256	128	>256	>256	>256	128	128	>256	>256	8	64	0.03	>256	128	64	>256	16	>512	>512
KP38-1	128	>512	512	512	256	512	512	128	128	512	512	2	64	0.06	512	128	128	>256	1	>512	>512
KP38-2	128	>512	512	512	256	512	512	128	128	512	512	2	64	0.06	512	128	128	>256	1	>512	>512

a ATM, aztreonam; FOF, fosfomycin; ETP, ertapenem; CAZ, ceftazidime; FEP, cefepime; SCF, cefoperazone-sulbactam; FOX, cefoxitin; LVX, levofloxacin; CIP, ciprofloxacin; AMK, amikacin; TET, tetracycline; TGC, tigecycline; MH, minocycline; CST, colistin; CTX, cefotaxime; MEM, meropenem; IPM, imipenem; GEN, gentamicin; SXT, trimethoprim-sulfamethoxazole; PRL, piperacillin; and PRL/TZP, piperacillin-tazobactam.

b mg/L.

### Identification of Antimicrobial Resistance Genes and Virulence Genes

According to the WGS data, antimicrobial resistance genes and virulence genes among the 16 ST11-KL64 CRKP isolates are determined and showed in Figure 2A. All the isolates harbored aminoglycosides (*rmtB*), chloramphenicol (*catA2*), fosfomycin (*fosA*), quinolones (*qnrS1*), sulfonamides (*sul2*), tetracyclines [*tet(A)*], and β-lactams (*bla*_CTX-M-65_, *bla*_KPC-2_, *bla*_SHV-12_, and *bla*_TEM-1B_) resistance genes. Aminoglycoside resistance gene *aadA2* was observed in 15 CRKP isolates but only absent in isolate KP5. Trimethoprim resistance gene *dfrA14* was also found in all CRKP isolates except for KP38-1. Moreover, the analysis of quinolone resistance-determining regions (QRDRs) revealed three amino acid mutations associated with fluoroquinolone resistance, including S83I and D87G in GyrA and S80I in ParC, in all the 16 CRKP isolates. Additionally, all the 16 ST11-KL64 CRKP isolates contained aerobactin (*iucABCD* and *iutA*) and hypermucoviscosity (*rmpA2*) virulence genes, representing a potential CR-hvKP phenotype. The regulator of mucoid phenotype gene (*rmpA*) and *peg-344* were detected in all CRKP isolates except for KP35, KP37, KP38-2, and KP45.

### Genetic Features of Virulence Plasmids and *bla*_KPC-2_-Bearing Plasmids

**Figure 4:**
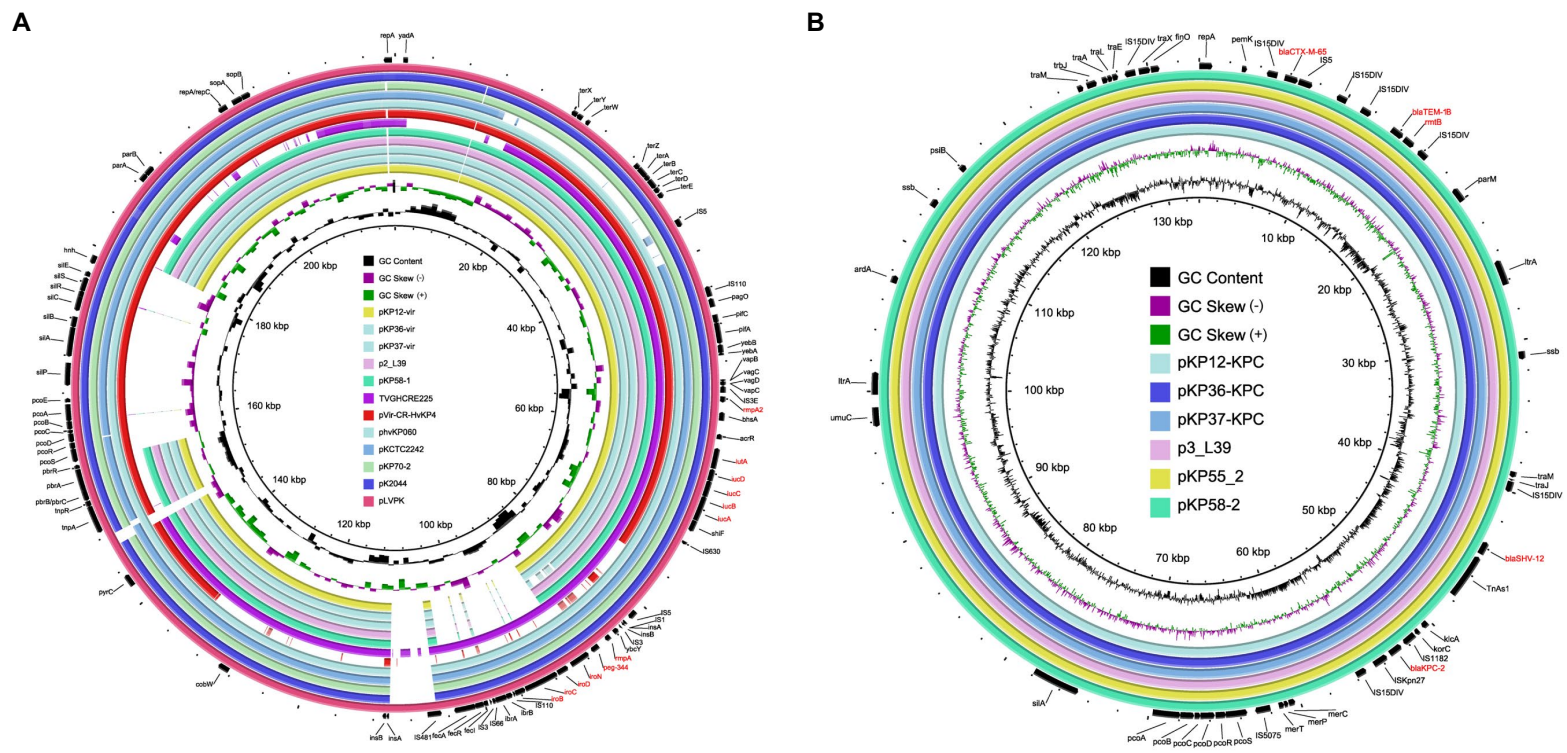
Genetic comparison of virulence plasmids and *bla*_KPC-2_-bearing plasmids recovered from *K. pneumoniae* KP12, KP36, and KP37 with similar plasmids in the NCBI database, respectively. **(A)** Alignment of virulence plasmids pKP12-vir, pKP36-vir, and pKP37-vir with p2_L39 (CP033955), pKP58-1 (CP041374), TVGHCRE225 (CP023723), pVir-CR-HvKP4 (MF437313), phvKP060 (CP034776), pKCTC2242 (CP002911), pKP70-2 (MF398271), pK2044 (AP006726), and pLVPK (AY378100). pLVPK was used as the reference plasmid. Virulence genes are highlighted in red. **(B)** Alignment of *bla*_KPC-2_-bearing plasmid pKP12-KPC, pKP36-KPC, and pKP37-KPC with p3_L39 (CP033956), pKP55_2 (CP055296), and pKP58-2 (CP041375). p3_L39 was used as the reference plasmid. Antimicrobial resistance genes are highlighted in red.

**Figure 5:**
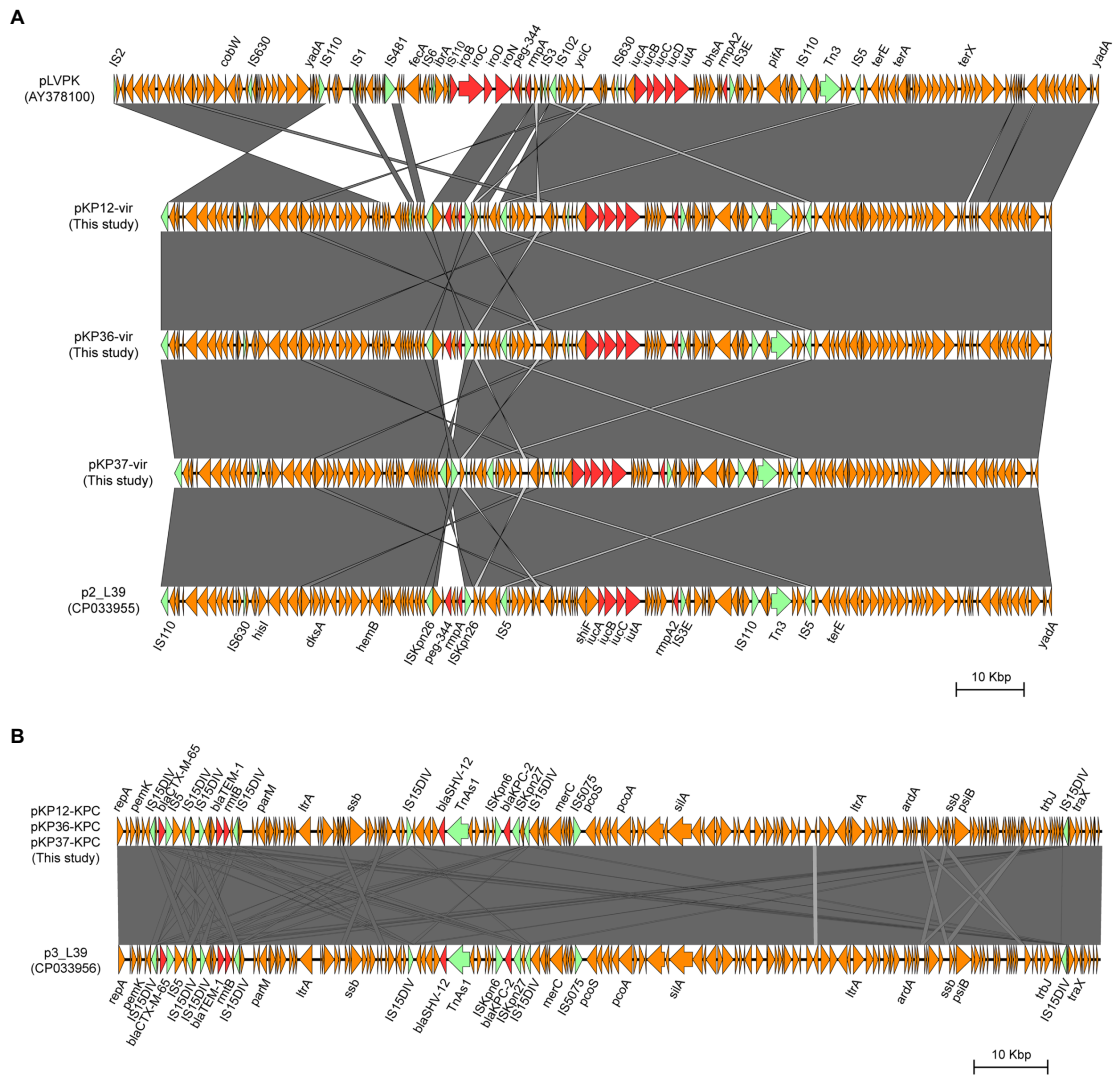
Structural comparison of the genetic context of virulence genes **(A)** and *bla*_KPC-2_ gene **(B)** in representative plasmids. The arrows represent coding sequences (red arrows, antimicrobial resistance genes or virulence genes; green arrows, mobile elements). Shading denotes the regions with high homology (>95% nucleotide identity).

To investigate the genetic features of virulence plasmids and *bla*_KPC-2_-bearing plasmids, *K. pneumoniae* KP12, KP36, and KP37 were further subjected to whole-genome sequencing using the Oxford Nanopore MinION platform. Plasmid sequence analysis confirmed that all the three *K. pneumoniae* isolates harbored an IncFIB/IncHI1B type virulence plasmid and an IncFII/IncR type *bla*_KPC-2_-bearing plasmid (Figure 4). Interestingly, *iroBCDN* with IS*110* transposase on the upstream, located in the same transposon IS*3* with *rmpA* and *peg-344* in pLVPK, were absent in KP12, KP36, and KP37 (Figure 5A). It is worth noting that *rmpA* and *peg-344* were located between two IS*Kpn26* transposons in pKP12-vir and pKP36-vir, but the two virulence genes were further missing in pKP37-vir. According to these results, we hypothesize that IS*110* might be responsible for the deletion of *iroBCDN*, and then forming a new transposon IS*Kpn26* carrying *rmpA* and *peg-344* from the classical virulence plasmid pLVPK after this losing event.

Plasmids pKP12-KPC, pKP36-KPC, and pKP37-KPC exhibited a high level of homology (99% coverage and 99% identity) with other *bla*_KPC-2_-bearing plasmids, i.e., the 133,772-bp IncFII/IncR plasmid p3_L39 recovered from *K. pneumoniae* strain L39_2 in China. Plasmids pKP12-KPC, pKP36-KPC, and pKP37-KPC also harbored several antimicrobial resistance genes, i.e., *bla*_CTX-M-65_, *bla*_SHV-12_, *bla*_TEM-1B_, and *rmtB* (Figure 5B). Comparison of genetic surroundings of *bla*_KPC-2_ gene indicated that it was flanked by a similar core structure, IS*Kpn27*-*bla*_KPC-2_-IS*Kpn6*. Another multi-drug resistance region consisted of *bla*_CTX-M-65_, *bla*_TEM-1B_, and *rmtB* genes and five mobile genetic elements on these *bla*_KPC-2_-bearing plasmids.

## Discussion

The worldwide spread of carbapenem-resistant *K. pneumoniae*, associated with considerable morbidity and mortality, poses a severe threat to public health. ST11 was found to be the most predominant epidemic clone of CRKP strains in China, which has aroused considerable attention recently due to the scenarios for convergence of resistance and hypervirulence determinants in a single strain (Wyres et al., 2020a). Therefore, surveillance and tracking of high-risk clones of CRKP and understanding their clinical importance are critical. PFGE and MLST, as well-known genotyping methods, can monitor and control the spread of nosocomial pathogens (Singh et al., 2006). However, traditional molecular typing techniques are limited in distinguishing minor genomic differences between closely related strains involved in an outbreak. With the extensive use of next-generation sequencing, WGS analysis easily distinguishes minor differences between clones and is widely used for pathogen identification and tracking the rules underlying pathogen spread (Klemm et al., 2018; Schurch et al., 2018). This study investigated the origin and transmission pattern of KPC-2-producing ST11-KL64 CRKP outbreaks. The genetic features of virulence plasmids and *bla*_KPC-2_-bearing plasmids were further investigated by comparative genomic analysis.

During the study period, 47 *K. pneumoniae* isolates were identified as CRKP, and three dominant PFGE clusters were observed. Among them, 16 CRKP isolates harbored the *bla*_KPC-2_ gene, representing a potential clonal spread due to their similar PFGE patterns. *In silico* MLST analysis identified all the 16 KPC-2-producing CRKP isolates as ST11, which is the most predominant sequence type of KPC-2-producing CRKP in China and has been reported worldwide, including America, Europe, and Asia (Baraniak et al., 2011; Jiang et al., 2015; Zhan et al., 2017; Gu et al., 2018; Ko, 2019; Spencer et al., 2019; Zhou et al., 2020; Jin et al., 2021; Yang et al., 2021). Moreover, all the 16 KPC-2-producing CRKP isolates belonged to the serotype KL64, which differs from those reported previously, such as KL1, KL2, and KL62 (Feng et al., 2018; Gu et al., 2018). However, an infection caused by KPC-2-producing ST11-KL64 CRKP was frequently reported recently (De Campos et al., 2018; Jia et al., 2019; Ruan et al., 2020a; Zhang et al., 2020b; Zhou et al., 2020; Jin et al., 2021; Yang et al., 2021). Zhou et al. (2020) reported that patients infected by ST11-KL64 CRKP had a significantly higher mortality rate than those infected by other CRKP. In this study, four out of 16 patients died of ST11-KL64 CRKP infection. These findings suggested that KL64 clone of CRKP was closely related to high pathogenicity and transmissibility, and continuous monitoring should be taken to prevent further dissemination.

Following cgSNP-based phylogenetic analysis, 16 KPC-2-producing ST11-KL64 CRKP isolates could be divided into two clades, suggesting two independent transmission events. Some patients in the two clades showed overlaps in time frames or hospital stay units that may have contributed to *K. pneumoniae* dissemination among patients. The index patient of transmission 1 was Pa 32, and the infection was transmitted to Pa 33, Pa 35, Pa 36, and Pa 37 represented in clade 1. Pa 33, Pa 35, Pa 36, and Pa 37 shared months of overlapping ICU stay with Pa 32, and the five isolates from these inpatients were sampled in ICU and displayed considerable genomic similarity. CRKP was detected in the urine and blood culture of Pa 38 and Pa 45 after they were transferred from ICU to neurosurgery. A separate transmission route of KPC-2-producing CRKP infection was identified in Pa 12, Pa 15, Pa 18, and Pa 19. The sampling time of the above four inpatients was close, suggesting that a transmission event occurred within a short period. The spread of ST11 CRKP among different departments or different wards of the same department in the hospital is relatively expected, which has been reported frequently (Jiang et al., 2015; Gu et al., 2018; Sui et al., 2018; Lu et al., 2020; Zhang et al., 2020b). Our data suggested two independent outbreaks of KPC-2-producing ST11-KL64 CRKP isolates in the ICU and the respiratory ward, respectively, between 2016 and 2018. These results confirmed the easy transfer of ST11 CRKP, and practical strategies must be implemented to control the outbreak and avoid nosocomial transmission.

In this study, all the isolates of 16 ST11-KL64 CRKP harbored *bla*_KPC-2_ and were multidrug-resistant. In addition to carbapenemases, several extended-spectrum β-lactamase (ESBL) resistance genes, including *bla*_CTX-M-65_, *bla*_SHV-12_, and *bla*_TEM-1B_, were identified in the 16 ST11-KL64 CRKP isolates. This finding indicated that all isolates contained multiple ESBL genes, which is in agreement with the reports on the co-occurrence of *bla*_CTX-M_, *bla*_SHV_, and *bla*_TEM_ in *K. pneumoniae* strains (Baraniak et al., 2011; Jiang et al., 2015; Gu et al., 2018; Sui et al., 2018). Therefore, more attention should be paid to the *K. pneumoniae* isolates, identified as epidemic ST11 clone and co-harbored carbapenemases and ESBLs in China. Besides the β-lactams resistance genes, other resistance genes that were found to be present in the majority of 16 ST11-KL64 CRKP isolates included *rmtB*, *catA2*, *fosA*, *qnrS1*, *sul2*, *tet*(A), *aadA2* (detectable in 15 isolates), and *dfrA14* (detectable in 15 isolates), conferring resistance to aminoglycosides, chloramphenicol, fosfomycin, quinolone, sulphonamides, tetracycline, aminoglycoside, and trimethoprim, respectively. Undoubtedly, the presence of resistance genes allows the *K. pneumoniae* isolates to survive the barrage of antimicrobial agents used in treating infections. Fortunately, tigecycline and colistin were still effective *in vitro*, which suggested that the above two antimicrobials could be valuable treatment choices against ST11-KL64 CRKP infections.

Several virulence factors have been characterized as contributing to the hypervirulence phenotype of *K. pneumoniae*, but are not limited to the mucoid regulators *rmpA* and *rmpA2* and the aerobactin synthesis cluster *iucABCD/iutA* (Russo and Marr, 2019; Choby et al., 2020). The *rmpA* and *rmpA2* genes have both been considered determinants controlling the capsular polysaccharide (CPS) biosynthesis, representing the hypermucoviscous phenotype. The aerobactin has been appreciated as the predominant siderophore system in the hvKP. It has previously been common that ST11 *K. pneumoniae* is a widely disseminated multidrug-resistant clonal lineage, including carbapenems, but not hypervirulent. However, a fetal outbreak in five patients caused by an ST11 carbapenem-resistant *K. pneumoniae* strain, which turned into hvKP by acquiring a roughly 170 kb pLVPK-like plasmid, was reported in China (Gu et al., 2018). In this study, all the 16 KPC-2-producing K64-ST11 CRKP isolates harbored hypermucoviscous phenotype regulators and aerobactin synthesis, showed not only resistance to antimicrobials but also hypervirulent phenotype. Alongside the plasmid-borne ESBL and carbapenemase genes, our data revealed a high prevalence of ST11-KL64 CR-hvKPs carrying the IncHI1B/IncFIB virulence plasmids with *iro* locus deletion. Clinically, the KPC-2-producing ST11-KL64 CRKP isolates carrying the *rmpA* and *rmpA2* genes exhibited enhanced environmental survival and caused more severe infection than classic ST11 KP strains, leading to high mortality (Zhou et al., 2020). This finding is consistent with our study, which showed that all the four dead patients (Pa 1, Pa 33, Pa36, and Pa38) harbored *rmpA* and *rmpA2* genes in ST11-KL64 CRKP isolates. These results revealed the hypervirulence nature of those isolates, and targeted surveillance is urgently needed. Further genomic epidemiology and evolutionary analysis at the national scale are warranted to understand the genetic basis and evolution characteristics of the wide dissemination of carbapenem-resistant hypervirulent ST11-KL64 *K. pneumoniae* in China.

During the two nosocomial CPKP outbreaks, we had implemented infection prevention and control measures to control the outbreaks in the ICU and respiratory. Several measures were implemented to eliminate the nosocomial CPKP infection. First, we implemented stringent isolation procedures for the CRKP-infected patients and limited the persons who came to contact with these patients. Second, we performed equipment disinfection and periodic environmental cleansing. Third, all the hospital staff contacted with patients carrying CRKP should wear medical gloves and contagion gowns and enforced hand hygiene once the operation was finished. Finally, the ST11-KL64 CRKP nosocomial infection was successfully eliminated and ended in April 2018.

In conclusion, our study identified the emergence of a high-risk clone of KPC-2-producing ST11-KL64 CRKP isolates in a clinical setting. Our results clearly showed that WGS could reveal the two transmission scenarios caused by these CRKP isolates. Due to the acquisition of multiple plasmid-borne antimicrobial resistance and virulence genes, these isolates have presented a significant challenge for public health. The placement of adequate infection control measures is necessary to prevent their further dissemination in nosocomial settings.

## Data Availability Statement

The datasets presented in this study can be found in online repositories. The names of the repository/repositories and accession number(s) can be found in the article/Supplementary Material.

## Author Contributions

ZR, JZ, and XX designed the experiments. YK, QS, and HC performed the experiments. ZR and YK analyzed the data. YK, ZR, and MD wrote the manuscript. All authors contributed to the article and approved the submitted version.

## Funding

This study was supported by the National Natural Science Foundation of China (81871696 and 82072342) and the Zhejiang Provincial Medical and Health Science and Technology Plan (2021KY943).

## Conflict of Interest

The authors declare that the research was conducted in the absence of any commercial or financial relationships that could be construed as a potential conflict of interest.

## Publisher’s Note

All claims expressed in this article are solely those of the authors and do not necessarily represent those of their affiliated organizations, or those of the publisher, the editors and the reviewers. Any product that may be evaluated in this article, or claim that may be made by its manufacturer, is not guaranteed or endorsed by the publisher.
