# Supplementary material for: Transmission Dynamics of Carbapenem-Resistant Klebsiella pneumoniae Sequence Type 11 Strains Carrying Capsular Loci KL64 and rmpA/rmpA2 Genes
Source: Front Microbiol. 2021 Oct 7;12:736896. doi: 10.3389/fmicb.2021.736896 (PMC8529244; doi:10.3389/fmicb.2021.736896)
Supplement: Supplementary file 2 [file Table_2.DOCX]

**Table S2.** **MICs of the remaining 31 CRKP isolates to different antimicrobials.**

| Isolate | **ATM^a^** | **FOF** | **ETP** | **CAZ** | **FEP** | **SCF** | **FOX** | **LVX** | **CIP** | **AMK** | **TET** | **TGC** | **MH** | **CST** | **CTX** | **MEM** | **IPM** | **GEN** | **SXT** | **PRL** | **PRL/TZP** |
| --- | --- | --- | --- | --- | --- | --- | --- | --- | --- | --- | --- | --- | --- | --- | --- | --- | --- | --- | --- | --- | --- |
| KP7 | 128^b^ | 16 | >256 | 128 | 256 | >256 | >256 | 32 | 64 | >256 | 256 | 4 | 32 | 0.03 | 128 | 128 | 128 | 1 | 16 | >512 | >512 |
| KP11 | 128 | 512 | 64 | 128 | 32 | >256 | 64 | 16 | 32 | >256 | 2 | 0.25 | 8 | 0.06 | 64 | 16 | 16 | >256 | 16 | >512 | >512 |
| KP14 | 128 | 32 | >256 | 128 | 256 | >256 | >256 | 32 | 64 | >256 | 256 | 2 | 32 | 0.03 | 128 | 128 | 32 | 1 | 16 | >512 | >512 |
| KP17 | 128 | 16 | >256 | >256 | 256 | >256 | >256 | 128 | 128 | >256 | >256 | 8 | 64 | 0.06 | >256 | 128 | 128 | >256 | 16 | >512 | >512 |
| KP22 | 128 | >512 | 64 | 128 | 64 | 256 | 128 | 16 | 16 | >256 | 8 | 2 | 32 | 0.06 | 128 | 128 | 16 | >256 | 16 | >512 | 512 |
| KP23 | 128 | 16 | >256 | >256 | 256 | >256 | >256 | 64 | 128 | >256 | >256 | 4 | 64 | 0.03 | >256 | 128 | 128 | >256 | 16 | >512 | >512 |
| KP24 | 128 | 8 | >256 | >256 | 256 | >256 | >256 | 64 | 128 | >256 | >256 | 4 | 64 | 0.03 | >256 | 128 | 32 | >256 | 16 | >512 | >512 |
| KP25 | 128 | >512 | 8 | 32 | 16 | 128 | 128 | 32 | 16 | >256 | 8 | 4 | 32 | 0.06 | 32 | 8 | 8 | 64 | 16 | 256 | 256 |
| KP27 | 128 | >512 | 256 | 64 | 256 | 512 | 256 | 16 | 16 | 512 | 2 | 2 | 4 | 0.03 | 512 | 16 | 16 | 256 | 16 | 256 | 256 |
| KP28 | 128 | 32 | >256 | >256 | 256 | >256 | >256 | 64 | 128 | >256 | >256 | 4 | 32 | 0.03 | >256 | 128 | 128 | >256 | 0.5 | >512 | >512 |
| KP31 | 128 | 32 | >256 | >256 | 512 | >256 | >256 | 64 | 128 | >256 | >256 | 2 | 32 | 0.25 | >256 | 128 | 64 | >256 | 16 | >512 | >512 |
| KP40 | 128 | 64 | >256 | 32 | 512 | >256 | >256 | 16 | 4 | >256 | >256 | 1 | 8 | 1 | >256 | 128 | 64 | 1 | 8 | >512 | >512 |
| KP42 | 128 | >512 | >256 | >256 | 512 | >256 | >256 | 128 | 128 | 4 | >256 | 4 | 32 | 0.063 | >256 | 128 | 128 | >256 | 16 | >512 | >512 |
| KP46 | 128 | 16 | 32 | >256 | 32 | 256 | 256 | 2 | 4 | >256 | 2 | 0.5 | 4 | 0.03 | 256 | 16 | 8 | >256 | 16 | >512 | >512 |
| KP47 | 128 | >512 | 256 | >256 | 256 | >256 | >256 | 16 | 32 | >256 | 256 | 4 | 32 | 0.125 | >256 | 128 | 128 | >256 | 16 | >512 | 512 |
| KP48 | 128 | >512 | 256 | 128 | 256 | 512 | 512 | 16 | 32 | 512 | 4 | 1 | 4 | 0.03 | 512 | 16 | 32 | >256 | 16 | >512 | 512 |
| KP49 | 128 | 64 | 256 | 128 | 512 | >256 | 256 | 32 | 32 | 1 | 8 | 4 | 32 | 0.06 | 256 | 64 | 128 | 128 | 16 | >512 | 512 |
| KP50 | 128 | 64 | 512 | 256 | 512 | 512 | 512 | 16 | 32 | 512 | 2 | 1 | 4 | 0.03 | 512 | 16 | 32 | >256 | 16 | >512 | 512 |
| KP51 | 128 | >512 | 512 | 128 | 512 | 512 | 512 | 128 | 128 | 512 | 512 | 8 | 32 | 0.125 | 512 | 128 | 128 | >256 | 16 | >512 | 512 |
| KP52 | 64 | >512 | 2 | 128 | 32 | 256 | >256 | 4 | 2 | >256 | 4 | 2 | 32 | 0.03 | 128 | 128 | 128 | >256 | 16 | >512 | 512 |
| KP53 | 128 | >512 | >256 | >256 | 128 | 128 | 256 | 8 | 16 | >256 | 2 | 1 | 8 | 0.25 | >256 | 128 | 128 | >256 | 16 | >512 | >512 |
| KP54 | 32 | >512 | 8 | 4 | 32 | 128 | 64 | 0.5 | 8 | 4 | 8 | 2 | 32 | 8 | 64 | 128 | 128 | >256 | 1 | >512 | >512 |
| KP55 | 128 | >512 | 256 | 128 | 256 | 512 | 512 | 16 | 32 | 512 | 4 | 1 | 4 | 0.03 | 512 | 128 | 32 | >256 | 16 | >512 | >512 |
| KP56 | 128 | 64 | >256 | >256 | 256 | >256 | >256 | 128 | 128 | >256 | 4 | 4 | 4 | 0.03 | >256 | 128 | 128 | >256 | 16 | >512 | >512 |
| KP57 | 128 | 2 | 2 | 8 | 2 | 32 | 16 | 16 | 0.125 | 1 | >256 | 2 | 64 | 0.03 | 8 | 32 | 16 | 0.25 | 16 | 256 | 128 |
| KP58 | 128 | >512 | 128 | 256 | 256 | >256 | 128 | 16 | 32 | >256 | 256 | 2 | 16 | 0.03 | >256 | 32 | 32 | >256 | 4 | >512 | 256 |
| KP59 | 128 | 16 | 256 | 512 | 256 | 512 | 256 | 16 | 32 | 512 | 4 | 2 | 0.25 | 4 | 512 | 32 | 32 | >256 | 1 | >512 | >512 |
| KP62 | 128 | >512 | 2 | 32 | 32 | 128 | 16 | 4 | 16 | 2 | 256 | 1 | 8 | 0.03 | 32 | 2 | 2 | 128 | 16 | >512 | 512 |
| KP63 | 128 | >512 | 2 | 64 | 32 | 128 | 16 | 4 | 16 | 2 | 128 | 1 | 8 | 0.03 | 32 | 2 | 4 | 128 | 16 | >512 | 512 |
| KP65 | 128 | 64 | >256 | 128 | >256 | >256 | >256 | 128 | 128 | >256 | >256 | 8 | 64 | 0.03 | >256 | 128 | 64 | >256 | 16 | >512 | >512 |
| KP86 | 128 | 32 | >256 | 128 | >256 | >256 | >256 | 128 | 128 | >256 | >256 | 2 | 64 | 0.03 | >256 | 128 | 64 | >256 | 16 | >512 | >512 |

^a^ ATM, aztreonam; FOF, fosfomycin; ETP, ertapenem; CAZ, ceftazidime; FEP, cefepime; SCF, cefoperazone-sulbactam; FOX, cefoxitin; LVX, levofloxacin; CIP, ciprofloxacin; AMK, amikacin; TET, tetracycline; TGC, tigecycline; MH, minocycline; CST, colistin; CTX, cefotaxime; MEM, meropenem; IPM, imipenem; GEN, gentamicin; SXT, trimethoprim-sulfamethoxazole; PRL, piperacillin; PRL/TZP, piperacillin-tazobactam.

^b^: mg/L.
